# Gestational diabetes in the pregnant Omani population in relation to their fatty acid intake and levels

**DOI:** 10.1186/s12884-026-09027-y

**Published:** 2026-05-01

**Authors:** Mohammed Al Sinani, Mark Johnson, Michael Crawford, Mohammed Al Maqbali

**Affiliations:** 1 Department of Nutrition, Al-Buraimi Hospital, Ministry of Health, Al Buraimi, Oman; 2 Fatima College of Health Sciences, Al Ain, United Arab Emirates; 3 Department of Metabolism, Digestion and Reproduction Imperial College London, 369 Fulham Road, SW10 9NH London, UK

**Keywords:** GDM, FAs, n-3 FAs, DHA, EPA, ArA

## Abstract

**Introduction:**

Low omega-3 Fatty acids (FAs) levels are associated with insulin resistance and upregulation of adiponectin and women with Gestational diabetes mellitus (GDM) have lower n-3 FAs. The current study assesses the association between FAs intake and individual fatty acid levels and ratios in maternal and cord erythrocyte FAs’ profile GDM among sample of pregnant Arabic-speaking women in Oman.

**Methodology:**

This prospective cohort study was conducted among pregnant Omani women attending antenatal clinics in Al Buraimi, Oman, between 2019 and 2021. A subgroup of women diagnosed with gestational diabetes mellitus was compared with healthy pregnant controls. This analysis focuses on a subset of 56 women diagnosed with GDM compared to a control group of 101 healthy women. Seafood and the n-3 FAs intakes of women has been quantified by using a validated Food frequency questionnaire (FFQ). Maternal and cord blood FAs analysis of erythrocytes was carried out using the method of Folch et al.

**Results:**

DHA and Omega-3 index (n-3 index) intake in the GDM population was significantly lower than in control group (*p* = 0.031) and (*p* = 0.05). GDM women had lower levels of Arachidonic acid (ArA) and Docosahexaenoic acid (DHA) (*p* = 0.002), total n-3 FAs (*p* = 0.001), but a higher level of lignoceric acid (24:00), Eicosapentaenoic acid EPA In term of cord erythrocytes, GDM women was significantly lower Adrenic acid LA, ArA, DHA, Adrenic acid (AdA), total n-6 FAs and EPA (*p* = 0.021) compared to the women in the control group.

**Conclusions:**

GDM women have a lower n-3 FAs intake reflected in their lower levels of erythrocyte membrane DHA. These findings provide the rationale for a randomized controlled trial of DHA replacement in pregnant women with low DHA levels to test the hypothesis that DHA replacement reduces the risk of developing GDM.

## Introduction

GDM is a type of hyperglycaemia that develops during pregnancy either due to insulin deficiency or increased insulin resistance [1]. The prevalence of GDM is estimated to vary from 2% to 16% [2]. GDM is associated with adverse effects on the pregnancy, including fetal macrosomia, polyhydramnios, preterm labour, operative delivery, shoulder dystocia, stillbirth; in the perinatal period, neonatal respiratory distress, hypoglycaemia, and jaundice; and in the long-term for the child: obesity, lower Intelligence Quotient (IQ) and type 2 diabetes (T2D) and for the mother an increased risk of cardiovascular disease (CVD) and T2D [3, 4].

Risk factors for gestational diabetes mellitus include a family history of type 2 diabetes, previous GDM, older maternal age, raised body mass index, and certain ethnic backgrounds that are known to have a higher predisposition to insulin resistance. Furthermore, micronutrient deficiency has been linked with GDM onset [5]. The main factor responsible for more than 80% of GDM cases is obesity, where elevated proinflammatory cytokines/adipokines drive chronic inflammation and an increase in insulin resistance [6]. Thus, anti-inflammatory mechanisms may help to reduce GDM occurrence and improve the outcomes of pregnancies complicated by GDM.

Polyunsaturated fatty acids (PUFAs) have been recognised as having anti-inflammatory properties [6], improved insulin sensitivity and become a progressively intriguing focus for disease prediction and the mitigation of of various diseases, including CVD, T2D, inflammatory disorders, and certain types of cancer [7, 8].

In the human body, there are two essential FAs, LA (n-6) and alpha-linolenic acid ALA (n-3) that cannot be produced in the body and therefore must be acquired through the diet or supplements [9]. The dietary intake in most of Western society has significantly declined in n-3 FAs intake which could hypothetically stimulate the pathogenesis of numerous chronic conditions. It is well-recognised that high n-3 FAs levels and intake in women during pregnancy increases gestational age, reducing preterm delivery [10, 11], reducing the occurrence of children’s allergies [12, 13] and enhancing neurocognitive development [13].

Lower levels of omega-3 fatty acids (n-3 FAs) have been reported in women with gestational diabetes mellitus (GDM) [14, 15], In contrast, higher dietary intake and higher circulating levels of n-3 FAs have been associated with reduced insulin resistance, inflammation, and oxidative stress [16, 17].

Furthermore, in a randomized controlled trial (RCT) n-3 FAs supplements show reduction in GDM risk [18]. However, other RCT examine n-3 FAs effects on GDM reported no significantly impact plasma glucose levels, HOMA-B, QUICKI, or lipid profiles [17, 18].

Further work is needed to determine the importance of n-3 FAs in the occurrence of GDM and whether supplementation can improve insulin sensitivity. Further, it is unclear whether FAs levels or ratios might be useful screening tools to select those at high risk of GDM.

The current study assesses the association between FAs intake and levels in maternal erythrocytes and the occurrence of GDM among Omani pregnant women. Despite growing evidence linking fatty acid metabolism to insulin resistance and gestational diabetes mellitus (GDM), most existing studies have focused on dietary intake or plasma fatty acid concentrations and have been conducted predominantly in Western or East Asian populations. There is a paucity of data examining erythrocyte fatty acid composition, which reflects longer-term fatty acid status, in relation to GDM, particularly in Middle Eastern populations, where dietary patterns and metabolic risk profiles differ substantially.

This study is novel in that it simultaneously examines dietary omega-3 fatty acid intake and erythrocyte fatty acid profiles in both maternal and cord blood among pregnant Omani women, comparing those with and without GDM. To our knowledge, this is one of the first studies from the Gulf region to explore these associations using erythrocyte biomarkers, providing insight into longer-term fatty acid status in relation to GDM.

## Methods

This prospective cohort study investigates the relationship between FAs intake and levels and maternal depressive and anxiety symptoms and pregnancy outcomes among a sample of pregnant Omani women (N:302) [19]. The current analysis focuses on a subset of 56 women diagnosed with GDM compared to a control group of 101 healthy women, drawn from a total cohort of 302 participants. The Ethics and Research Committee Board of the Oman Ministry of Health approved the study protocol on 1 July 2019 (MoH/CSR/19/9668).

The study’s inclusion and exclusion criteria, recruitment process, and assessment methods have been previously described in detail [20]. Briefly, the inclusion criteria include: (i) aged between 18 and 45 years of age; (ii) 8–12 weeks gestation of single pregnancies; (iii) expecting to continue prenatal health care in the antenatal clinic at Al Buraimi Hospital; (iv) agreeing to participate in the study and are residents of Al Buraimi, Oman. Pregnant women who were non-Omani, or with severe health conditions (e.g. cancer, heart disease, and any infectious disease such as Hepatitis C and B carriage, human immunodeficiency virus (HIV), cirrhosis or other chronic liver diseases were excluded. The diagnosis of GDM was made based on standard diagnostic criteria, which have been described previously [21].

Sociodemographic variables and maternal data were recorded using a health and lifestyle questionnaire and medical notes. As part of routine antenatal care, all participants were prescribed standard prenatal multivitamin supplements in accordance with national guidelines. Omega-3 fatty acid supplementation was not routinely prescribed, and detailed information on specific multivitamin formulations or compliance was not available.

Dietary intake of omega-3 fatty acids was assessed using a short, previously validated food frequency questionnaire (FFQ) designed by Kuratko [22] designed to estimate habitual intake of fish and long-chain omega-3 fatty acids, including eicosapentaenoic acid (EPA) and docosahexaenoic acid (DHA). The FFQ was translated into Arabic using a forward–backward translation process and piloted for clarity and cultural appropriateness among pregnant Omani women prior to data collection.

Participants reported their usual frequency of fish and seafood consumption. Estimated intakes of EPA and DHA were calculated by multiplying reported consumption frequency by standard portion sizes and published nutrient composition values. The omega-3 index intake estimate was derived from the combined EPA and DHA intake. Although the FFQ was not formally validated in an Omani population, it has been widely used in pregnancy studies and was selected to minimize participant burden. This has been acknowledged as a study limitation.

The lipid extraction in this research project for maternal and cord erythrocytes samples was done following the Folch et al. [23] approach, and FAs compositions were analysed by capillary gas chromatography [24, 25].

Maternal and umbilical cord blood samples were collected, and erythrocytes were separated by centrifugation and washed with isotonic saline. Total lipids were extracted from erythrocytes using the method described by Folch et al. Briefly, lipids were extracted using a chloroform–methanol (2:1, v/v) solution, followed by phase separation. Fatty acids were transmethylated to fatty acid methyl esters (FAMEs) and analysed by capillary gas chromatography using authenticated standards for identification and quantification. Fatty acid composition was expressed both as a percentage of total identified fatty acids and as absolute concentrations (mg/mL of erythrocytes). This dual expression was used to provide complementary information on relative fatty acid distribution and absolute fatty acid content.

The results presented are based on the participants who met our inclusion criteria. Written consent was obtained from all participants. Throughout this manuscript, the term ‘fatty acid levels’ refers specifically to erythrocyte fatty acid composition, unless otherwise stated, while ‘fatty acid intake’ refers to dietary intake estimated from the food frequency questionnaire.

Data analysis was performed using the Statistical Package for the Social Sciences (SPSS), Version 21, developed by IBM Corp., Chicago, Illinois, USA. Descriptive statistics were used to describe pregnant women’s socioeconomic, demographic, and psychological data. For continuous variables, the results were expressed as means, and standard deviations (SD), while categorical variables were presented as frequencies and percentages. The relationship between FAs level profile and GDM among the study samples were matched using the Mann-Whitney U test. The primary objective of this analysis was to compare dietary intake and erythrocyte fatty acid profiles between women with and without gestational diabetes mellitus. Therefore, non-parametric group comparison tests were applied due to the non-normal distribution of fatty acid variables. Logistic regression analysis was not performed, as the study was not designed to develop a predictive model for gestational diabetes risk.

## Results

Table 1 shows the demographic and socioeconomic of the pregnant Omani GDM women participating in the study. Most of the GDM women were from the age group between 30 and 40 years (46.4%). Approximately 51% of the study samples had a university degree or higher. Most of the study samples (68%) were homemakers and 71% had planned their pregnancy. At booking, 30% were overweight, and 50% were obese.55% did no exercise, 28% reported exercising for 30 min at least twice a week and.17% followed a regular exercise routine of 30 min 3/week. There were no statistically significant differences between GDM women and the control group regarding demographic and socioeconomic variables.

**Table 1:** Sociodemographic and gestational characteristics among pregnant Omani women N (157)

	GDM 56		Control 101				GDM 56		Control 101		
	*N*	%	*N*	%	*P*		*N*	%	*N*	%	*P*
**Age (Y)**					0.31	**Planned pregnancy**					0.91
< 20	1	1.8	5	5		Yes	40	71.4	73	72.3	
20–30	22	39.3	48	47.5		No	16	28.6	28	27.7	
30–40	26	46.4	42	41.6		**Physical Activity**					0.94
> 40	7	12.5	6	5.9		No	31	55.4	51	50.5	
**Educational level**					0.30	30 min for at least 2/ week	16	28.6	32	31.7	
Basic Education	4	7.1	14	13.9		30 min for at least 3/week	3	5.4	7	6.9	
Secondary Education	29	51.8	42	41.6		30 min for at least 5/week	6	10.7	11	10.9	
Degree or higher	23	41.1	45	44.6		**Parity**					0.19
**Occupational Status**					0.85	1	8	14.3	27	26.7	
Employed	18	32.1	31	30.7		2	14	25	20	19.8	
Unemployed	38	67.9	70	69.3		3	34	60.7	54	53.5	
**Annual household Income**					0.42	**BMI at registration**					0.07
Less than 500 OR	24	42.9	41	40.6		Underweight	4	7.1	8	7.9	
500 OR – 1000 OR	25	44.6	39	38.6		Normal	7	12.5	30	29.7	
Above 1000	7	12.5	21	20.8		Overweight	17	30.4	29	28.7	
						Obesity	28	50.	34	33.7	

Information on physical activity during pregnancy was available for the study participants. The majority of women with gestational diabetes mellitus (GDM) reported low levels of physical activity, with more than half reporting no regular exercise during pregnancy. There were no statistically significant differences in reported exercise frequency between women with GDM and the control group.

The fish intake by portion per week and the median (interquartile range) nutritional intake of DHA and EPA of the study participants are shown in Table 2. The fish consumption of 1–2 portions per week and more than two portions of fish per week in the group of women with GDM was reported by 49 of the pregnant women (87.5%), and only 7pregnant women (12.5%) reported that they did not eat fish at all. In terms of n-3 FAs DHA and EPA intake, as shown in Table 2, women with a GDM (n:56) revealed a significantly lower median intake of DHA and n-3 index = 183 vs. 220; *p* < 0.03), ( n-3 index, = 325 vs. 375 ; *p* < 0.05), compared to the healthy control group. The median intake of DHA and n-3 index were significantly linked with the incidence of GDM among women in the study (*p* = 0.031) and (*p* = 0.05), respectively. Dietary intake of FAs, DHA and n-3 index in the GDM population was significantly lower than in healthy pregnant women (*p* = 0.031) and (*p* = 0.05).

**Table 2:** Association between fish and omega-3 FAs intake and pregnancy and neonatal outcomes of the Omani pregnant women in the study (*n* = 157)

Variables		Fish Intake								Omega-3 Fatty Acids Intake					
		Non/week		1–2/week		≥ 3/week				DHA (mg)		EPA (mg)		*N* 3 index (mg/day)	
**GDM**	n	n	%	n	%	n	*%*	(*df*) χ^2^	*p*	Median	p	Median	p	Median	*p*
Women with GDM	56	7	12.5	20	35.7	29	51.8	0.2	0.905	183	**0.031**	126	0.134	325	**0.05**
Healthy control	101	11	12.8	35	35.4	55	51.8			220		143		375	

Table 3 reports the median proportions of maternal erythrocyte FAs levels for the women with GDM and the control group. Maternal erythrocytes from women with gestational diabetes mellitus (GDM) contained significantly higher levels of palmitic acid (PA), adrenic acid (AdA), the long-chain omega-6 to long-chain omega-3 fatty acid ratio (Lc n-6/Lc n-3), and the omega-6 to omega-3 fatty acid ratio (n-6/n-3) (*p* < 0.001) compared with the healthy control group. Conversely, women with GDM had lower erythrocyte levels of arachidonic acid (ArA), total omega-3 fatty acids (total n-3), docosahexaenoic acid (DHA), the omega-3 index (n-3 index), and the combined (arachidonic acid + docosahexaenoic acid) to monounsaturated fatty acid ratio ((AA + DHA)/MUFA) than women in the control group.

**Table 3:** Associations between maternal FAs levels (FAs % of total FAs in RBCs) of pregnant women with and without GDM

Fatty acid(% of total FAs in RBCs)	Control (n=101)	GDM (n=56)	
	Median (Min-Max)	Median (Min-Max)	p
16:00	24.12 (20.1-39.28)	26.55 (19.13-32.67)	0.008
24;00	3.62 (2.1-5.94)	4.175 (2.06-6.13)	<.001
∑SFA	45.51 (37.64-58.19)	47.6 (38.81-56.92)	0.015
18:1ω9	10.49 (6.78-13.56)	11.28 (6.12-14.02)	0.042
20:2ω6	0.72 (0.1-1.89)	0.965 (0.11-1.92)	0.033
20:4ω6	13.8 (8-17.9)	12.78 (7.4-16.98)	0.025
22:4ω6	1.43 (0.26-3.27)	1.7 (0.67-3.09)	0.045
20:3 ω3	0.5 (0.14-2.76)	0.33 (0.1-1.79)	0.014
22:6 ω3	5.9 (1.29-9.02)	2.98 (1.18-7)	0.002
Lc ω3	9.42 (3.14-14.56)	6.285 (3.27-12.89)	0.001
∑ω3	9.97 (4.37-15.2)	6.5 (3.87-13.97)	<.001
AA/LA	2.04 (0.7-4.32)	1.675 (0.74-3.12)	0.01
ω6/ω3	2.65 (1.63-6.99)	3.67 (1.86-7.21)	<.001
Lc ω6/Lc ω3	2.0872 (1.07-6.4)	3.0088 (1.09-5.74)	0.002
22:5/22:4ω6	0.41 (0.06-3.33)	0.2 (0.06-1.2)	0.003
ω3 Index (EPA+DHA)	6.96 (2.22-12.72)	4.505 (1.87-9.59)	0.002
(AA+DHA)/MUFA	1.07 (0.52-1.52)	0.92 (0.59-1.37)	<.001

*GDM* gestational diabetes mellitus, *FA* fatty acid, *LA* linoleic acid, *ALA* α-linolenic acid, *ArA* arachidonic acid, *AdA* adrenic acid, *DHA* docosahexaenoic acid, *EPA* eicosapentaenoic acid, *DPA* docosapentaenoic acid, *MUFA* monounsaturated fatty acids, *PUFA* polyunsaturated fatty acids, *SFA* saturated fatty acids, *n-3* omega-3, *n-6* omega-6

The absolute levels of maternal erythrocyte FAs (mg/ml) from women with GDM vs. healthy controls are shown in Table 4. Women with GDM had significantly lower levels of ArA (*p* = 0.025), total n-3 (*p* = 0.001), DPA, DHA (*p* = 0.004), n-3 index (*p* = 0.002), and (AA + DHA)/MUFA ratios, but a higher Lc n-6/Lc n-3 and n-6/n-3 ratio (p = < 0.001).

**Table 4:** Associations between maternal FAs levels in absolute quantities (mg/ml) of RBCs in pregnant women with and without GDM

Fatty acid profile (mg/mL)	control group (*n* = 101)	GDM (*n* = 56)	
	Median (Min-Max)	Median (Min-Max)	*p*
16:00	0.33(0.261–0.550)	0.368(0.261–0.450)	**0.029**
24;00	0.049(0.029–0.080)	0.058(0.027–0.086)	**< ** **0.001**
20:2ω6	0.010(0.001–0.027)	0.013(0.001–0.025)	**0.046**
20:4ω6	0.152(0.088–0.197)	0.141(0.081–0.187)	**0.025**
20:3 ω3	0.007(0.002–0.039)	0.005(0.001–0.023)	**0.006**
20:5 ω3	0.014(0.003–0.056)	0.013(0.000-0.052)	**0.027**
22:6 ω3	0.078(0.017–0.126)	0.039(0.015–0.098)	**0.004**
Lc ω3	0.124(0.041–0.285)	0.078(0.044–0.174)	**< ** **0.001**
∑ω3	0.129(0.053–0.208)	0.082(0.048–0.183)	**< ** **0.001**
AA/LA	1.685(0.550–3.397)	1.377(0.582–2.639)	**0.01**
ω6/ω3	2.494(1.513–6.871)	3.544(1.800-7.008)	**<** ** 0.001**
Lc ω6/Lc ω3	1.846(1.000-5.800)	2.691(1.000-5.250)	**0.002**
22:5/22:4ω6	0.323(0.049–2.821)	0.161(0.044–1.015)	**0.003**
ω3 Index (EPA + DHA)	0.096(0.029–0.178)	0.059(0.024–0.134)	**0.002**
(AA + DHA)/MUFA	0.928(0.439–1.309)	0.799(0.503–1.190)	**0.001**

*GDM* gestational diabetes mellitus, *FA* fatty acid, *LA* linoleic acid, *ALA* α-linolenic acid, *ArA* arachidonic acid, *AdA* adrenic acid, *DHA* docosahexaenoic acid, *EPA* eicosapentaenoic acid, *DPA* docosapentaenoic acid, *MUFA* monounsaturated fatty acids, *PUFA* polyunsaturated fatty acids, *SFA* saturated fatty acids, *n-3* omega-3, *n-6* omega-6

Table 5 shows the median cord erythrocyte FAs levels for women with and without GDM. The following cord RBC FAs levels were significantly higher in terms of SA and lignoceric acid (24:00), SFA, but a significantly lower in terms of LA, ArA, DDA, AdA and total n-6 FAs. Moreover, women in the GDM group had lower cord FAs in erythrocytes with regard to EPA (*p* = 0.021), and (AA + DHA)/MUFA ratio compared to the control group. Table 6 reports cord erythrocyte FAs in terms of absolute quantities (mg/ml) for the women with GDM and the healthy control group. The total cord FAs content in terms of erythrocytes in the GDM group had pointedly elevated levels of stearic acid (18:00) and total saturated fat, but a significantly lower level of LA, ArA, DDA, AD and total n-6 FAs. Furthermore, women in the GDM group had lower content of cord FAs in erythrocytes with regard to EPA and the (AA + DHA)/MUFA ratio.

**Table 5:** Associations between cord blood FAs levels (FAs % of total FAs in RBCs) of pregnant women with and without GDM

Fatty acid (% of total FAs in RBCs)	Control (*n* = 53)	GDM (*n* = 40)	
	Median (Min-Max)	Median (Min-Max)	*p*
18:00	17.21 (10.79–21.34)	17.985 (12.64–20.71)	**0.035**
∑SFA	52.41 (42.03–63.26)	56.13 (44.92–66.05)	**0.033**
18:2ω6	3.2 (1.54–4.73)	2.865 (1.2–4.53)	**0.045**
20:3ω6	1.47 (0.74–3.9)	3.5 (0.6–4.11)	**0.002**
20:4ω6	14.8 (9.62–18.76)	13.785 (9.1-19.01)	**0.028**
22:2ω6	0.65 (0.05–1.26)	0.51 (0.01–1.08)	**0.012**
22:4ω6	2.9 (1.18–4.7)	1.985 (1.03–4.87)	**0.018**
∑ω6	25.47 (16.43–31.48)	24.52 (16.45–33.81)	**0.044**
20:3 ω3	0.48 (0.08–1.88)	0.33 (0.07–1.58)	**0.021**
20:5 ω3	0.61 (0.21–1.9)	0.505 (0.16–1.63)	**0.047**
AA/DHA	3.35 (1.33–7.21)	3.09 (1.2–6.03)	**0.049**
(AA + DHA)/MUFA	1.35 (0.73–2.36)	1.21 (0.66–2.3)	**0.038**

**Table 6:** Associations between cord blood FAs levels in absolute quantities (mg/ml) of RBCs in pregnant women with and without GDM

Fatty acid profile (mg/mL)	Control group (*n* = 53)	GDM (*n* = 40)	
	Median (Min-Max)	Median (Min-Max)	*p*
18:00	0.220(0.138–0.273)	0.230(0.162–0.265)	**0.035**
∑SFA	0.671(0.538–0.810)	0.718(0.575–0.845)	**0.033**
18:2ω6	0.041(0.020–0.061)	0.037(0.015–0.058)	**0.045**
20:3ω6	0.019(0.010–0.050)	0.045(0.008–0.053)	**0.002**
20:4ω6	0.207(0.135–0.263)	0.193(0.127–0.267)	**0.028**
22:2ω6	0.008(0.001–0.016)	0.007(0.000-0.014)	**0.012**
22:4ω6	0.037(0.015–0.060)	0.025(0.013–0.062)	**0.018**
∑ω6	0.346(0.223–0.423)	0.333(0.225–0.461)	**0.042**
20:3 ω3	0.006(0.001–0.024)	0.004(0.001–0.020)	**0.022**
20:5 ω3	0.008(0.003–0.024)	0.006(0.002–0.021)	**0.047**
22:5/22:4ω6	0.260(0.081–1.419)	0.416(0.067–1.568)	**0.047**
(AA + DHA)/MUFA	1.438(0.784–2.516)	1.274(0.711–2.450)	**0.031**

## Discussion

The current study assesses the association between FAs intake and levels in a sample of pregnant Arabic-speaking women women with and without GDM in Oman. Maternal and fetal FAs metabolism may be affected by GDM, impacting fetal growth and development [26]. In many studies, differences in FAs metabolism have been observed between mothers with and without GDM [27].

Due to the 120-day lifespan of erythrocytes, their compositional data offers an integrated view of diet, lifestyle, genetics, and metabolism, making them a valuable indicator of long-term nutritional status compared to plasma or serum, which are more reflective of short-term variations.These findings suggest that erythrocyte fatty acid profiles may have potential utility as biomarkers to identify women at higher risk of developing gestational diabetes mellitus, rather than as diagnostic screening tools for hyperglycaemia. Such markers could help inform early risk stratification and guide future preventive or interventional studies. In addition, the lower levels of DHA observed in women who developed GDM and the evidence that DHA can enhance insulin sensitivity, supports a trial of DHA replacement in those with low DHA levels to assess its effect on GDM rates.

The current study investigates FAs levels using percentage of total FAs and absolute quantities. Given the high level of inconsistency in individuals’ intake and metabolic rates. FAs proportions reflect the relative significance of specific FAs compared to the total FAs composition, while absolute quantities measure FAs independently of others. Although expressing FAs in absolute quantities is recommended, percentages are often preferred in studies linking erythrocyte FAs profiles to pregnancy outcomes [24]. FAs ratios can provide insight into nutritional deficiencies or metabolic imbalances that may contribute to GDM [28].

This study shows that women with GDM had lower levels of DHA, n-3 index, and ArA compared to the control group and an elevated proportion of saturated FAs, n-6 FAs, and n-6/n-3 FAs ratio. This finding is consistent with earlier observational studies [26, 28]. Studies have revealed that women with GDM tend to have higher saturated FAs (SFA) and lower unsaturated FAs (USFA) than their healthy counterparts [26]. This shift towards SFAs has been linked with insulin resistance, a key feature of GDM [26]. However, studies that have examined subclasses of SFAs yielded different findings. For example, studies involving Chinese women revealed a beneficial association between MA and PA levels and GDM in comparison to healthy women [29, 30]. Others found opposing associations between odd-chain HA, PA, and even full-chain SFAs with GDM [31]. Similar results have been replicated in the study of T2D patients [32] and those with cardiovascular diseases [33]. Moreover, Chinese studies have confirmed these findings by establishing a correlation between elevated levels of long-chain SFA and an increased likelihood of developing GDM [34]. Metabolic irregularities and higher saturated fat levels, as observed in our GDM group, influence the production of n-3 and n-6 FAs from their parent FAs, LA and ALA and the activity of the enzymes responsible for essential fatty acid (EFA) metabolism is influenced by both insulin and adiposity [35].

Women with GDM had low n-3 FAs levels, specifically DHA and EPA [26]. A meta-analysis highlighted the potential role of n-3 supplementation in reducing insulin resistance in these women [36]. Moreover, the anti-inflammatory properties of DHA and EPA are known to decrease systemic inflammation and insulin resistance [37]. DHA reduce inflammation by inhibiting cytokine synthesis and promoting anti-inflammatory mediators [38]. Additionally, these FAs can enhance cell membrane fluidity, optimizing insulin receptor function and promoting better insulin sensitivity [39, 40]. N-3 FAs, including DHA, may have a direct significance in the development of GDM [28]. Low DHA may compromise the body’s ability to regulate adiponectin and synthesise DHA-derived anti-inflammatory agents, which act to inhibit TNFalpha secretion and upregulate adiponectin production. Low levels of DHA may affect the body’s ability to regulate adiponectin, leading to reduced production of anti-inflammatory agents and increased release of TNFalpha [41]. Although α-linolenic acid (ALA) is the dietary precursor of long-chain omega-3 fatty acids, its conversion to eicosapentaenoic acid (EPA) and docosahexaenoic acid (DHA) is limited in humans and is further reduced in states of insulin resistance. In the present study, dietary assessment focused on preformed EPA and DHA intake, and fatty acid status was evaluated using erythrocyte fatty acid composition, which reflects long-term exposure to biologically active long-chain omega-3 fatty acids. This approach minimises reliance on endogenous conversion of ALA and is particularly relevant in the context of gestational diabetes mellitus.

Women with GDM exhibit significantly higher levels of the parent n-6 FAs, linoleic acid (LA), but without a corresponding increase in its metabolites [26]. Typically, LA is converted to arachidonic acid (ArA) through elongase and desaturase enzyme activity [25]. However, the elevated LA/ArA ratio in GDM suggests a disruption in this conversion process, indicating potential deficiencies in these enzymes. This metabolic irregularity is unique to GDM and points to specific alterations in FAs metabolism associated with the condition [26].

We found lower levels of the beneficial MUFAs such as oleic acid (OA). OA has anti-inflammatory properties, promotes lipolysis, enhances Lpl activity, regulates n-3 FAs metabolism, and reduces proinflammatory cytokines [42]. Research has indicated that MUFAs positively impact insulin sensitivity and glucose metabolism [26]. Arbo et al. [43] had previously investigated the relationship between insulin sensitivity and changes in FAs metabolism through increased desaturase enzyme activity. OA supplementation has shown benefits in lipid and glucose metabolism and favourable gene regulation associated with inflammation reduction [44]. However, in the current study, FA levels were measured in early pregnancy suggesting that the changes in the FA profile were present before conception and therefore either have a causative role or are secondary to the already abnormal metabolic state which predisposes to the development of GDM.

In the current study, GDM women had higher ratios of n-6/n-3 FAs when compared to the healthy group. This is consistent with previous studies showing that the diet of obese individuals is typically low in n-3 FAs and high in n-6 FAs [28]. A lower dietary intake of ALA, the parent n-3 FAs, and low consumption of synthesised FAs may cause significantly lower n-3 levels. The n-6/n-3 FAs ratio plays a critical role in regulating inflammation, a key factor in GDM. An elevated n-6/n-3 ratio is associated with increased production of pro-inflammatory eicosanoids, worsening systemic inflammation and insulin resistance [28] The imbalance in this ratio seen in GDM patients fosters a pro-inflammatory state, contributing to disease progression.

In women with GDM, 1 g of n-3 FAs supplements daily for six weeks significantly decreased inflammatory markers and improved pregnancy outcomes [18]. The FAs supplementation improved fasting plasma glucose levels and decreased the levels of markers of inflammation markers (C-reactive protein) and of oxidative stress (malondialdehyde), as well as being associated with a lower rate of newborn hospitalisations, compared to the placebo [18].

The mechanisms responsible for the anti-inflammatory effects of n-3 FAs include modifying cell membrane FAs composition and inhibiting the pro-inflammatory transcription factor NF-κB activation [45]. And although providing pregnant women with n-3 FAs supplements for six weeks did not significantly impact fasting plasma glucose levels, it did reduce HOMA-IR through a reduction in serum insulin levels [37]. However, another study found that taking 800 mg/d of DHA during the middle stage of pregnancy had no effect on pre-eclampsia or GDM [46].

The current study shows that women in the GDM group had lower LA, ArA, total n-6 FAs, and total n-3 FAs in erythrocytes and (AA + DHA)/MUFA ratio compared to the control group. These findings are consistent with other studies that reported the levels of ArA and DHA were lower in women with GDM compared to those without [47, 48]. Further, the results are similar to those of a meta-analysis that showed mothers with GDM had lower cord blood levels of total n-6, ArA, total n-3 and DHA [49]. These differences may be because GDM affects placental FAs transport, particularly DHA [50]. Indeed, mothers with GDM had lower levels of placental FAs transport proteins, resulting in reduced DHA levels in cord blood [50]. This study did not comprehensively assess gestational weight gain or detailed dietary variables such as total energy intake, macronutrient composition, fiber intake, or fruit and vegetable consumption. Therefore, residual confounding by lifestyle and broader dietary factors cannot be excluded. In addition, dietary assessment focused primarily on omega-3 fatty acid intake, and future studies should incorporate more detailed dietary profiling. The sample size of women with gestational diabetes mellitus was relatively small, reflecting the exploratory nature of this subgroup analysis. Although this may limit statistical power and generalisability, the study provides novel data on erythrocyte fatty acid profiles in an understudied population. Larger prospective studies are required to confirm these findings and further explore their clinical relevance. Future studies with larger sample sizes are warranted to apply multivariable logistic regression models to evaluate the independent contribution of fatty acid intake and status to gestational diabetes risk.

## Conclusion

This study presents evidence to support the hypothesis that women diagnosed with GDM have a unique profile of FAs in early pregnancy, which is significantly different from that of healthy pregnant women. The study indicates that GDM women have substantially higher levels of n-6 FAs and lower levels of n-3 FAs. These findings provide unique evidence that can be used to develop a tool for predicting GDM risk in early pregnancy that can be applied in a clinical setting.

## Data Availability

All the data are available from the corresponding author upon reasonable request.
